# 2-Methyl-6-nitro-2*H*-indazole

**DOI:** 10.1107/S1600536811019374

**Published:** 2011-05-28

**Authors:** Li Long, Bing-Ni Liu, Mo Liu, Deng-Ke Liu

**Affiliations:** a School of Environmental and Chemical Engineering, Tianjin Polytechnic University, Tianjin 300160, People’s Republic of China; b Tianjin Institute of Pharmaceutical Research, Tianjin, 300193, People’s Republic of China

## Abstract

In the title compound, C_8_H_7_N_3_O_2_, the mol­ecular skeleton is almost planar with a maximum deviation of 0.0484 (9) Å for the methyl C atom. In the crystal, weak inter­molecular C—H⋯N and C—H⋯O hydrogen bonds help to establish the packing.

## Related literature

For the synthesis, see: Sorbera *et al.* (2006 ▶); Balardi *et al.* (1997 ▶). For related structures, see: Qi *et al.* (2010 ▶); Chen *et al.* (2009 ▶). For applications of indazole derivatives, see, for example: Li *et al.* (2008 ▶).
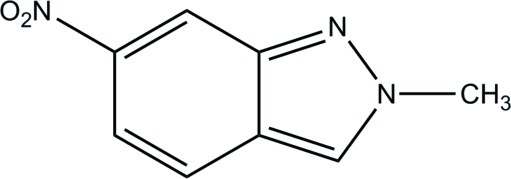


## Experimental

### 

#### Crystal data

C_8_H_7_N_3_O_2_
*M* *_r_* = 177.17
Monoclinic, 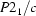

*a* = 3.793 (3) Å
*b* = 12.200 (8) Å
*c* = 16.675 (11) Å
β = 95.722 (9)°
*V* = 767.7 (9) Å^3^
*Z* = 4
Mo *K*α radiation
μ = 0.12 mm^−1^
*T* = 113 K
0.40 × 0.20 × 0.10 mm

#### Data collection

Rigaku Saturn724 CCD diffractometer
Absorption correction: multi-scan (*CrystalClear*; Rigaku/MSC, 2005 ▶) *T*_min_ = 0.956, *T*_max_ = 0.989
7726 measured reflections
1802 independent reflections
1317 reflections with *I* > 2σ(*I*)
*R*_int_ = 0.039

#### Refinement

*R*[*F* ^2^ > 2σ(*F* ^2^)] = 0.035
*wR*(*F* ^2^) = 0.092
*S* = 1.03
1802 reflections
119 parameters
H-atom parameters constrained
Δρ_max_ = 0.28 e Å^−3^
Δρ_min_ = −0.23 e Å^−3^

### 

Data collection: *CrystalClear* (Rigaku/MSC, 2005 ▶); cell refinement: *CrystalClear*; data reduction: *CrystalClear*; program(s) used to solve structure: *SHELXS97* (Sheldrick, 2008 ▶); program(s) used to refine structure: *SHELXL97* (Sheldrick, 2008 ▶); molecular graphics: *SHELXTL* (Sheldrick, 2008 ▶); software used to prepare material for publication: *CrystalStructure* (Rigaku/MSC, 2005 ▶).

## Supplementary Material

Crystal structure: contains datablocks global, I. DOI: 10.1107/S1600536811019374/cv5096sup1.cif

Structure factors: contains datablocks I. DOI: 10.1107/S1600536811019374/cv5096Isup2.hkl

Supplementary material file. DOI: 10.1107/S1600536811019374/cv5096Isup3.cdx

Supplementary material file. DOI: 10.1107/S1600536811019374/cv5096Isup4.cml

Additional supplementary materials:  crystallographic information; 3D view; checkCIF report

## Figures and Tables

**Table 1:** Hydrogen-bond geometry (Å, °)

*D*—H⋯*A*	*D*—H	H⋯*A*	*D*⋯*A*	*D*—H⋯*A*
C2—H2⋯N2^i^	0.95	2.52	3.446 (2)	164
C7—H7⋯O2^ii^	0.95	2.56	3.500 (2)	169
C8—H8*A*⋯O2^iii^	0.98	2.61	3.549 (2)	161
C8—H8*B*⋯O1^ii^	0.98	2.51	3.491 (2)	174

Symmetry codes: (i) 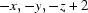; (ii) 
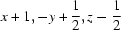
; (iii) 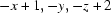.

**Fig. 1.**
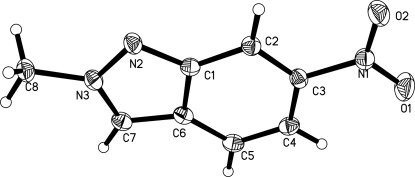
The molecular structure of (I), with the atom-numbering scheme and 50% probability displacement ellipsoids.

## supplementary crystallographic information

### Comment

Some derivatives of indazole are important intermediates in the synthesis of drugs (Li *et al.* 2008). Herewith we report the crystal structure of the tile compound (I), which is an intermediate in the synthesis of the antitumor agent pazopanib.

In (I) (Fig. 1), the bond lengths and angles are normal and comparable with those reported for related compounds (Qi *et al.*, 2010; Chen *et al.*, 2009). Rings N2/N3/C7/C6/C1 and C1—C6 are almost coplanar forming a dihedral angle 1.08 (7) °. The indazole ring system is almost planar with the maximal deviation of 0.0118 (7) Å for atom N3. Nitro group is twisted at 0.93 (16)° from the plane of the attached indazole ring system.

In the crystal structure, weak intermolecular C—H···N and C—H···O hydrogen bonds (Table 1) help to establish the packing.

### Experimental

The title compound was prepared according to Sorbera *et al.* (2006) and Balardi *et al.* (1997). 6-Nitro-1*H*-indazole (2 g) was dissolved in regurgitant dichloromethane (30 ml), then dimethyl sulfate (1.7 ml) and dimethyl sulfoxide (2 ml) were introduced. The mixture was heated to reflux and stayed for 12 h. The reaction mixture was washed with saturated sodium bicarbonate (10 ml) and then extracted with dichloromethane (20 ml), dried with sodium sulfate and evaporated, to give yellow solid (78% yield). Crystals suitable for X-ray analysis were obtained by slow evaporation of a dichloromethane.

### Refinement

H atoms were positioned geometrically, with C—H = 0.95 and 0.98 Å for indazole and methyl H, and refined as riding atoms, with *U*_iso_(H) = 1.2*U*_eq_(C) or 1.5*U*_eq_(C_methyl_).

### Crystal data

**Table:**

C_8_H_7_N_3_O_2_	*F*(000) = 368
*M**_r_* = 177.17	*D*_x_ = 1.533 Mg m^−^^3^
Monoclinic, *P*2_1_/*c*	Mo *K*α radiation, λ = 0.71073 Å
Hall symbol: -P 2ybc	Cell parameters from 2636 reflections
*a* = 3.793 (3) Å	θ = 2.1–28.0°
*b* = 12.200 (8) Å	µ = 0.12 mm^−^^1^
*c* = 16.675 (11) Å	*T* = 113 K
β = 95.722 (9)°	Prism, colourless
*V* = 767.7 (9) Å^3^	0.40 × 0.20 × 0.10 mm
*Z* = 4	

### Data collection

**Table:**

Rigaku Saturn724 CCD diffractometer	1802 independent reflections
Radiation source: rotating anode	1317 reflections with *I* > 2σ(*I*)
multilayer	*R*_int_ = 0.039
Detector resolution: 14.22 pixels mm^-1^	θ_max_ = 27.9°, θ_min_ = 2.1°
ω and φ scans	*h* = −4→4
Absorption correction: multi-scan (*CrystalClear*; Rigaku/MSC, 2005)	*k* = −15→16
*T*_min_ = 0.956, *T*_max_ = 0.989	*l* = −21→21
7726 measured reflections	

### Refinement

**Table:**

Refinement on *F*^2^	Primary atom site location: structure-invariant direct methods
Least-squares matrix: full	Secondary atom site location: difference Fourier map
*R*[*F*^2^ > 2σ(*F*^2^)] = 0.035	Hydrogen site location: inferred from neighbouring sites
*wR*(*F*^2^) = 0.092	H-atom parameters constrained
*S* = 1.03	*w* = 1/[σ^2^(*F*_o_^2^) + (0.05*P*)^2^] where *P* = (*F*_o_^2^ + 2*F*_c_^2^)/3
1802 reflections	(Δ/σ)_max_ = 0.001
119 parameters	Δρ_max_ = 0.28 e Å^−^^3^
0 restraints	Δρ_min_ = −0.23 e Å^−^^3^

### Special details

**Table:**

Geometry. All e.s.d.'s (except the e.s.d. in the dihedral angle between two l.s. planes) are estimated using the full covariance matrix. The cell e.s.d.'s are taken into account individually in the estimation of e.s.d.'s in distances, angles and torsion angles; correlations between e.s.d.'s in cell parameters are only used when they are defined by crystal symmetry. An approximate (isotropic) treatment of cell e.s.d.'s is used for estimating e.s.d.'s involving l.s. planes.
Refinement. Refinement of *F*^2^ against ALL reflections. The weighted *R*-factor *wR* and goodness of fit *S* are based on *F*^2^, conventional *R*-factors *R* are based on *F*, with *F* set to zero for negative *F*^2^. The threshold expression of *F*^2^ > σ(*F*^2^) is used only for calculating *R*-factors(gt) *etc*. and is not relevant to the choice of reflections for refinement. *R*-factors based on *F*^2^ are statistically about twice as large as those based on *F*, and *R*- factors based on ALL data will be even larger.

### Fractional atomic coordinates and isotropic or equivalent isotropic displacement parameters (Å^2^)

**Table:**

	*x*	*y*	*z*	*U*_iso_*/*U*_eq_	
O1	0.0001 (2)	0.40192 (7)	1.13521 (6)	0.0330 (3)	
O2	−0.1008 (2)	0.23042 (7)	1.15673 (5)	0.0285 (2)	
N1	0.0034 (2)	0.30444 (8)	1.11496 (6)	0.0204 (2)	
N2	0.2860 (2)	0.04933 (8)	0.90394 (6)	0.0191 (2)	
N3	0.4265 (2)	0.07413 (8)	0.83460 (6)	0.0189 (2)	
C1	0.2577 (3)	0.14834 (9)	0.93942 (7)	0.0164 (3)	
C2	0.1284 (3)	0.16936 (9)	1.01433 (7)	0.0172 (3)	
H2	0.0426	0.1128	1.0463	0.021*	
C3	0.1351 (3)	0.27717 (9)	1.03783 (7)	0.0172 (3)	
C4	0.2556 (3)	0.36516 (9)	0.99200 (7)	0.0197 (3)	
H4	0.2521	0.4380	1.0120	0.024*	
C5	0.3767 (3)	0.34458 (9)	0.91902 (7)	0.0202 (3)	
H5	0.4565	0.4026	0.8874	0.024*	
C6	0.3805 (3)	0.23462 (9)	0.89161 (7)	0.0175 (3)	
C7	0.4868 (3)	0.18162 (9)	0.82402 (7)	0.0197 (3)	
H7	0.5827	0.2147	0.7794	0.024*	
C8	0.5084 (3)	−0.01456 (9)	0.78106 (8)	0.0225 (3)	
H8A	0.6589	−0.0687	0.8114	0.034*	
H8B	0.6335	0.0150	0.7372	0.034*	
H8C	0.2879	−0.0497	0.7586	0.034*	

### Atomic displacement parameters (Å^2^)

**Table:**

	*U*^11^	*U*^22^	*U*^33^	*U*^12^	*U*^13^	*U*^23^
O1	0.0491 (6)	0.0212 (5)	0.0301 (5)	0.0001 (4)	0.0114 (4)	−0.0077 (4)
O2	0.0399 (5)	0.0249 (5)	0.0227 (5)	−0.0030 (4)	0.0126 (4)	0.0002 (4)
N1	0.0211 (5)	0.0207 (5)	0.0195 (5)	0.0007 (4)	0.0026 (4)	−0.0017 (4)
N2	0.0233 (5)	0.0198 (5)	0.0149 (5)	−0.0002 (4)	0.0058 (4)	0.0000 (4)
N3	0.0200 (5)	0.0216 (5)	0.0155 (5)	0.0003 (4)	0.0040 (4)	0.0004 (4)
C1	0.0147 (5)	0.0172 (5)	0.0171 (6)	0.0000 (4)	0.0004 (4)	0.0013 (4)
C2	0.0182 (6)	0.0172 (5)	0.0163 (6)	−0.0002 (4)	0.0021 (5)	0.0013 (4)
C3	0.0155 (6)	0.0203 (6)	0.0158 (6)	0.0016 (4)	0.0018 (5)	−0.0004 (5)
C4	0.0194 (6)	0.0161 (6)	0.0235 (6)	−0.0009 (4)	0.0010 (5)	0.0008 (5)
C5	0.0194 (6)	0.0189 (6)	0.0222 (6)	−0.0015 (5)	0.0026 (5)	0.0046 (5)
C6	0.0151 (6)	0.0208 (6)	0.0163 (6)	−0.0002 (4)	0.0008 (4)	0.0027 (5)
C7	0.0193 (6)	0.0218 (6)	0.0185 (6)	−0.0013 (5)	0.0036 (5)	0.0033 (5)
C8	0.0262 (6)	0.0234 (6)	0.0190 (6)	0.0016 (5)	0.0073 (5)	−0.0029 (5)

### Geometric parameters (Å, °)

**Table:**

O1—N1	1.2367 (14)	C3—C4	1.4194 (17)
O2—N1	1.2294 (14)	C4—C5	1.3662 (19)
N1—C3	1.4639 (17)	C4—H4	0.9500
N2—C1	1.3539 (16)	C5—C6	1.4178 (17)
N2—N3	1.3547 (15)	C5—H5	0.9500
N3—C7	1.3459 (17)	C6—C7	1.3934 (18)
N3—C8	1.4559 (16)	C7—H7	0.9500
C1—C2	1.4102 (18)	C8—H8A	0.9800
C1—C6	1.4265 (17)	C8—H8B	0.9800
C2—C3	1.3719 (17)	C8—H8C	0.9800
C2—H2	0.9500		
			
O2—N1—O1	122.63 (11)	C5—C4—H4	120.2
O2—N1—C3	119.20 (10)	C3—C4—H4	120.2
O1—N1—C3	118.17 (10)	C4—C5—C6	118.44 (11)
C1—N2—N3	103.23 (10)	C4—C5—H5	120.8
C7—N3—N2	114.58 (10)	C6—C5—H5	120.8
C7—N3—C8	126.47 (11)	C7—C6—C5	135.47 (11)
N2—N3—C8	118.91 (10)	C7—C6—C1	104.27 (11)
N2—C1—C2	126.80 (10)	C5—C6—C1	120.25 (11)
N2—C1—C6	111.69 (11)	N3—C7—C6	106.23 (11)
C2—C1—C6	121.50 (11)	N3—C7—H7	126.9
C3—C2—C1	115.41 (10)	C6—C7—H7	126.9
C3—C2—H2	122.3	N3—C8—H8A	109.5
C1—C2—H2	122.3	N3—C8—H8B	109.5
C2—C3—C4	124.70 (12)	H8A—C8—H8B	109.5
C2—C3—N1	118.07 (10)	N3—C8—H8C	109.5
C4—C3—N1	117.22 (10)	H8A—C8—H8C	109.5
C5—C4—C3	119.69 (11)	H8B—C8—H8C	109.5
			
C1—N2—N3—C7	−0.23 (12)	N1—C3—C4—C5	−179.08 (10)
C1—N2—N3—C8	177.69 (9)	C3—C4—C5—C6	−0.52 (16)
N3—N2—C1—C2	−179.34 (10)	C4—C5—C6—C7	−178.43 (12)
N3—N2—C1—C6	0.13 (12)	C4—C5—C6—C1	0.46 (16)
N2—C1—C2—C3	178.46 (10)	N2—C1—C6—C7	0.01 (12)
C6—C1—C2—C3	−0.96 (15)	C2—C1—C6—C7	179.51 (10)
C1—C2—C3—C4	0.92 (16)	N2—C1—C6—C5	−179.19 (9)
C1—C2—C3—N1	179.81 (9)	C2—C1—C6—C5	0.31 (16)
O2—N1—C3—C2	1.59 (15)	N2—N3—C7—C6	0.24 (12)
O1—N1—C3—C2	−178.04 (10)	C8—N3—C7—C6	−177.50 (10)
O2—N1—C3—C4	−179.44 (9)	C5—C6—C7—N3	178.87 (12)
O1—N1—C3—C4	0.93 (14)	C1—C6—C7—N3	−0.14 (11)
C2—C3—C4—C5	−0.19 (17)		

### Hydrogen-bond geometry (Å, °)

**Table:**

*D*—H···*A*	*D*—H	H···*A*	*D*···*A*	*D*—H···*A*
C2—H2···N2^i^	0.95	2.52	3.446 (2)	164
C7—H7···O2^ii^	0.95	2.56	3.500 (2)	169
C8—H8A···O2^iii^	0.98	2.61	3.549 (2)	161
C8—H8B···O1^ii^	0.98	2.51	3.491 (2)	174

Symmetry codes: (i) −*x*, −*y*, −*z*+2; (ii) *x*+1, −*y*+1/2, *z*−1/2; (iii) −*x*+1, −*y*, −*z*+2.
